# Help cure Parkinson’s disease: please don’t waste the Golden Year

**DOI:** 10.1038/s41531-018-0065-1

**Published:** 2018-09-25

**Authors:** Robert A. Hauser

**Affiliations:** University of South Florida, Tampa, FL USA

This isn’t a solicitation for a financial donation. But I am going to ask for something very valuable. It is in short supply and critical to our getting an approved therapy to slow progression of Parkinson’s disease (PD), and ultimately to reaching our shared goal of getting to a cure.

The discovery that aggregation of misfolded alpha-synuclein and propagation of this process through the autonomic and central nervous system lies at the heart of PD suggested many targets potentially amenable to therapeutic interventions.^1^ Enthusiasm is rightly running high that interruption of this process, or enhancement of mechanisms that clear toxic aggregates will slow or stop disease progression.

However, we still do not have validated biomarkers, such as a blood test, that allow us to assess disease progression and test these promising therapies in people with PD. Although several different clinical trial designs have been employed,^2–4^ the most common and straightforward methodology to test a promising disease modifying therapy is to assess its ability to slow progression of clinical signs and symptoms versus placebo over time in patients with early PD while they are not receiving symptomatic PD medications (e.g., levodopa, dopamine agonists, MAO-B inhibitors).

But we have a fundamental challenge! Individuals who are diagnosed with early PD based on some combination of bradykinesia (slow and small movement), rigidity (stiffness), and tremor (shaking), can only be followed without institution of symptomatic medication for about 6–12 months.^2^ After that time, a substantial proportion of patients will want or need symptomatic medication to relieve increasing signs and symptoms.

Ideally, we would like to be able to diagnose motor PD as early as possible and follow patients in a clinical trial of a potential disease modifying medication as long as possible to give us the best chance of identifying separation of rate of progression between those treated with active medication and those treated with placebo. If the time period over which we test the intervention is short, we reduce our ability to identify a difference between the intervention and placebo. If the time period over which we attempt to test the medication is too long, a substantial proportion of patients may require institution of symptomatic therapy and we lose our ability to monitor clinical disease progression during the observation period. Therefore, most trials of potential disease modifying medications in early PD follow patients for about 6 months to 1 year.

**Fig. 1:**
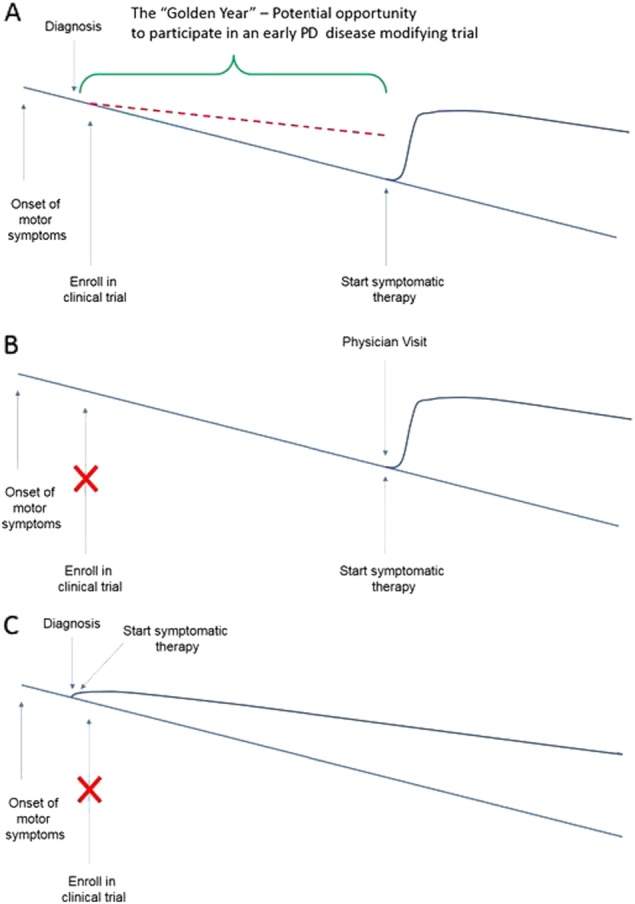
Relationship between time of diagnosis, need for symptomatic therapy and the opportunity for a patient to participate in an early PD disease modifying therapy trial. **a** The patient is diagnosed with early PD soon after the onset of motor symptoms and is enrolled in a disease modifying clinical trial. The “Golden Year” is the time from when a diagnosis of early PD can be made and the time when symptomatic therapy is needed. The red dashed line represents the potential disease slowing that might be associated with a disease modifying therapy. **b** The patient has waited to be evaluated until they need symptomatic therapy. They are no longer eligible to particpate in a disease modifying clinical trial and the Golden Year has been wasted. **c** The patient is diagnosed with early PD soon after the onset of motor symptoms but is immediately placed on symptomatic therapy. The patient is no longer eligible to participate in a disease modifying clinical trial and the Golden Year has been wasted

But patients with early PD who are available, and able and willing to enroll in a clinical trial, and whose PD symptoms are mild enough to go 6 months to 1 year before requiring symptomatic treatment are in short supply! To test promising potential disease modifying therapies, patients with early PD must be identified as early as possible and referred to centers where these clinical trials are being conducted before they go on symptomatic PD medications (Fig. 1a). Unfortunately, this process fails all too often. Patients frequently wait to make an appointment to undergo evaluation until their tremor, slowness, or rigidity are close to the point where they want symptomatic treatment. This is understandable because why see a doctor when you don’t need treatment? Nonetheless, at this point it is too late to participate in an early PD disease modifying trial (Fig. 1b). Additional unwanted delays may also occur due to time from setting the appointment to actually being seen. In another disappointing scenario, patients with early PD are diagnosed and immediately placed on symptomatic medication even though symptomatic treatment is not required (Fig. 1c). It may just be done reflexively and without consideration for the opportunity lost. Of course, we must also recognize that early introduction of symptomatic medication may be required in some cases if progression has been swift or if necessary to maintain employment.

The critical time of about one year from when the patient can be diagnosed with early PD based on mild classic motor features until they truly require symptomatic therapy can be considered their “Golden Year” for participation in disease modifying clinical trials. It is critical that care providers and patients don’t unknowingly waste this golden year. My pleas are these:

Care providers: When you are able to make a diagnosis of early PD, please don’t unnecessarily institute symptomatic therapy. Evaluate whether a clinical trial for early untreated PD patients is being conducted within a reasonable distance and discuss the possibility of participating in such a trial with the patient. At a minimum, let the patient know that there are early PD disease modifying trials being conducted and they may want to seek information on these trials before they decide to go on symptomatic therapy. Centers conducting such trials would be delighted to have the opportunity to discuss ongoing trials with the patient.

Patients: If you have new onset of tremor or slowness, or if a care provider says you may have PD, don’t wait until your condition worsens to the point that you feel you need treatment. Seek evaluation with a specialist as soon as possible. If a diagnosis of PD is made, ask about possible clinical trials. You may have to do your own internet search to find out more about currently enrolling trials. Useful websites include clinicaltrials.gov, michaeljfox.org, and parkinson.org. In addition, if a care provider makes a diagnosis of PD and wants to institute symptomatic therapy, consider seeking a second opinion from a center that conducts clinical trials before you start therapy. If you explain that you were recently diagnosed and interested in learning about clinical trials that center will often facilitate an appointment so as not to waste time.

Our ability to test promising new potential disease modifying therapies depends on both patients and health care providers understanding the critical value of the Golden Year in PD. I am asking individuals with early PD to consider participating in clinical trials, and the earlier, the better.

The journey to a cure will likely be incremental. If a medication does not work, we want to find out, discard it, and move on to other promising new therapies. Once we demonstrate that a therapy can slow disease progression, we will want to improve on it or find other therapies that can also slow progression and add to the effect. When we can slow disease progression sufficiently and can identify patients early enough, (even before the onset of slowness, stiffness, and tremor), the ultimate effect will be equivalent to having a cure.
